# Targeted Sequencing and Haplotype Analysis of Voltage-Gated Potassium Channel Genes Reveal a Potential Association of *KCNV2* Haplotypes with Antiseizure Medication Response in Turkish Patients with Epilepsy

**DOI:** 10.3390/ph19081193

**Published:** 2026-07-29

**Authors:** Kubra Cigdem Pekkoc-Uyanik, Zeynep Gizem Todurga-Seven, Erhan Rasit Agay, Hafize Uzun

**Affiliations:** 1 Department of Medical Biology, Faculty of Medicine, Haliç University, 34060 Istanbul, Turkey; kubrapekkoc@halic.edu.tr; 2 Department of Medical Pharmacology, Cerrahpasa Faculty of Medicine, Istanbul University-Cerrahpasa, 34098 Istanbul, Turkey; zeynep.seven@iuc.edu.tr (Z.G.T.-S.); erhanagay@gmail.com (E.R.A.); 3 Sinan Sipahi Family Health Centre, Republic of Turkey Ministry of Health, 34295 Istanbul, Turkey; 4 Department of Medical Biochemistry, Faculty of Medicine, Istanbul Atlas University, 34408 Istanbul, Turkey

**Keywords:** epilepsy, pharmacogenetics, *KCNA*, *KCNQ*, *KCNV*, next-generation sequencing, voltage-gated potassium channel genes, Kv

## Abstract

**Objective:** Voltage-gated potassium channel genes are among the most frequently implicated in epilepsy and antiseizure medication (ASM) response. In this pilot study, we aimed to identify rare and common variants by sequencing voltage-gated potassium channel (Kv) genes in epilepsy patients using ASM, and to reveal the potential drug responses of these variants. **Methods:** To investigate the role of genetic variants in Kv genes (*KCNQ1*, *KCNQ2*, *KCNQ3*, *KCNA1*, *KCNA2*, and *KCNV2*) in response to ASMs among 31 epilepsy patients, we used targeted next-generation sequencing (tNGS). Patients were classified as responders or persistent based on seizure control status. Selected variants in the genes were annotated, filtered, and analyzed for association with ASM response. **Results:** We identified 181 variants in the 6 channel genes, including missense, synonymous, intronic, UTR, and stop-gained variants. Six variants of uncertain significance (VUSs) were observed, including *KCNA2*c.*1314C>T, *KCNQ1*c.*976G>A, *KCNQ2* (c.2613G>T p.Arg871Ser and c.1148+62T>G), and *KCNQ3* (c.*6282A>G and c.*2860T>C)**.** Two novel variants were identified in our study group, *KCNA2*:c.*1314C>T and *KCNQ3*:c.*2860T>C. Both were located in the 3′ UTR region and classified as VUSs according to ACMG guidelines. In the *KCNV2* gene, the CG haplotype comprising rs7029012 and rs10967705 was observed more frequently in patients with drug-persistent epilepsy than in those with drug-responsive epilepsy (18.5% vs. 0.8%; χ^2^ = 6.756, *p* = 0.009, BH-FDR *q* = 0.036), suggesting a potential association with pharmacoresistant epilepsy. **Conclusions:** Our haplotype analysis suggests the potential pharmacogenetic contribution of the *KCNV2* gene to ASM response; however, these exploratory findings require validation in larger independent cohorts and functional studies.

## 1. Introduction

Epilepsy is a neurological disorder characterized by abnormal neuronal discharges in the brain and affects millions of individuals worldwide. Approximately 70–80% of epilepsy cases are estimated to have a genetic basis [1,2]. Advances in genetic research have improved syndrome classification, enabled the identification of novel therapeutic targets, and facilitated the development of precision medicine approaches. Notably, nearly one-quarter of epilepsy-associated genes encode ion channels [3].

Despite the availability of numerous antiseizure medications (ASMs), approximately 30% of patients remain drug-resistant, highlighting the need for more effective therapeutic strategies [4]. ASMs exert their antiepileptic effects through multiple molecular targets, including voltage-gated ion channels that regulate neuronal excitability [5].

Among these, Kv7.2/Kv7.3 potassium channels have emerged as promising therapeutic targets because of their prominent perisomatic and axonal expression and their ability to suppress neuronal hyperexcitability by limiting membrane depolarization [6,7,8]. Voltage-gated potassium (Kv) channels mediate K^+^ efflux during membrane repolarization and are essential for action potential generation, propagation, and synaptic transmission. Consequently, structural or functional alterations in these channels have been implicated in epilepsy and other neurological disorders [9].

The widespread application of targeted next-generation sequencing (tNGS) has accelerated the identification of epilepsy-associated genes, particularly those encoding potassium channel subunits [10]. Several Kv-related genes, including *KCNA1*, *KCNA2*, *KCNQ2*, *KCNQ3*, *KCNT1*, *KCNT2*, and *KCNMA1*, have been associated with epilepsy syndromes [11,12]. In particular, variants in *KCNQ1*, *KCNQ2*, *KCNQ3*, *KCNA1*, *KCNA2*, and *KCNV2* influence neuronal excitability by regulating action potential repolarization and may affect both epilepsy susceptibility and ASM response [13,14]. Disruption of channel structure or function caused by these variants can result in abnormal neuronal firing and diverse epileptic phenotypes.

Loss-of-function (LoF) variants in *KCNQ2* and *KCNQ3* are well-established causes of benign familial neonatal seizures and developmental and epileptic encephalopathies, whereas pharmacological activation of Kv7.2/Kv7.3 channels has demonstrated antiseizure efficacy [8,15]. Although the first-generation Kv7.2/Kv7.3 opener ezogabine was effective against focal-onset seizures (FOS), its clinical use was discontinued because of long-term adverse effects [15,16]. More recently, the selective Kv7.2/Kv7.3 opener XEN1101 has shown promise as a potential treatment for FOS [17]. Likewise, *KCNV2* variants have been associated with more severe epilepsy phenotypes through altered Kv2.1/Kv8.2 channel function [18]. Functional studies of *KCNA1* have further demonstrated that gain- and loss-of-function variants are associated with distinct clinical phenotypes and treatment responses, including enhanced sensitivity of certain gain-of-function variants to carbamazepine [19].

These findings highlight the potential of genotype-guided therapy in epilepsy [20]. Because genetic variation in Kv channel genes may contribute to interindividual differences in ASM response [9], we performed targeted next-generation sequencing of six candidate genes (*KCNQ1*, *KCNQ2*, *KCNQ3*, *KCNA1*, *KCNA2*, and *KCNV2*) in patients receiving ASM therapy to investigate their association with treatment response. Given that nearly one-third of patients remain resistant to ASMs [4], identifying pharmacogenetic determinants may improve patient stratification and support the development of personalized therapeutic approaches.

## 2. Results

### 2.1. Targeted Gene Sequencing Panel

The Targeted Gene Sequencing Panel of Kv Genes (*KCNA1*, *KCNA2*, *KCNQ1*, *KCNQ2*, *KCNQ3*, and *KCNV2*) in patients with epilepsy revealed both common and rare variants. We identified 181 variants in the six channel genes, including missense, synonymous, intronic, untranslated region (UTR), and stop-gained variants (Table 1).

**Figure 1:**
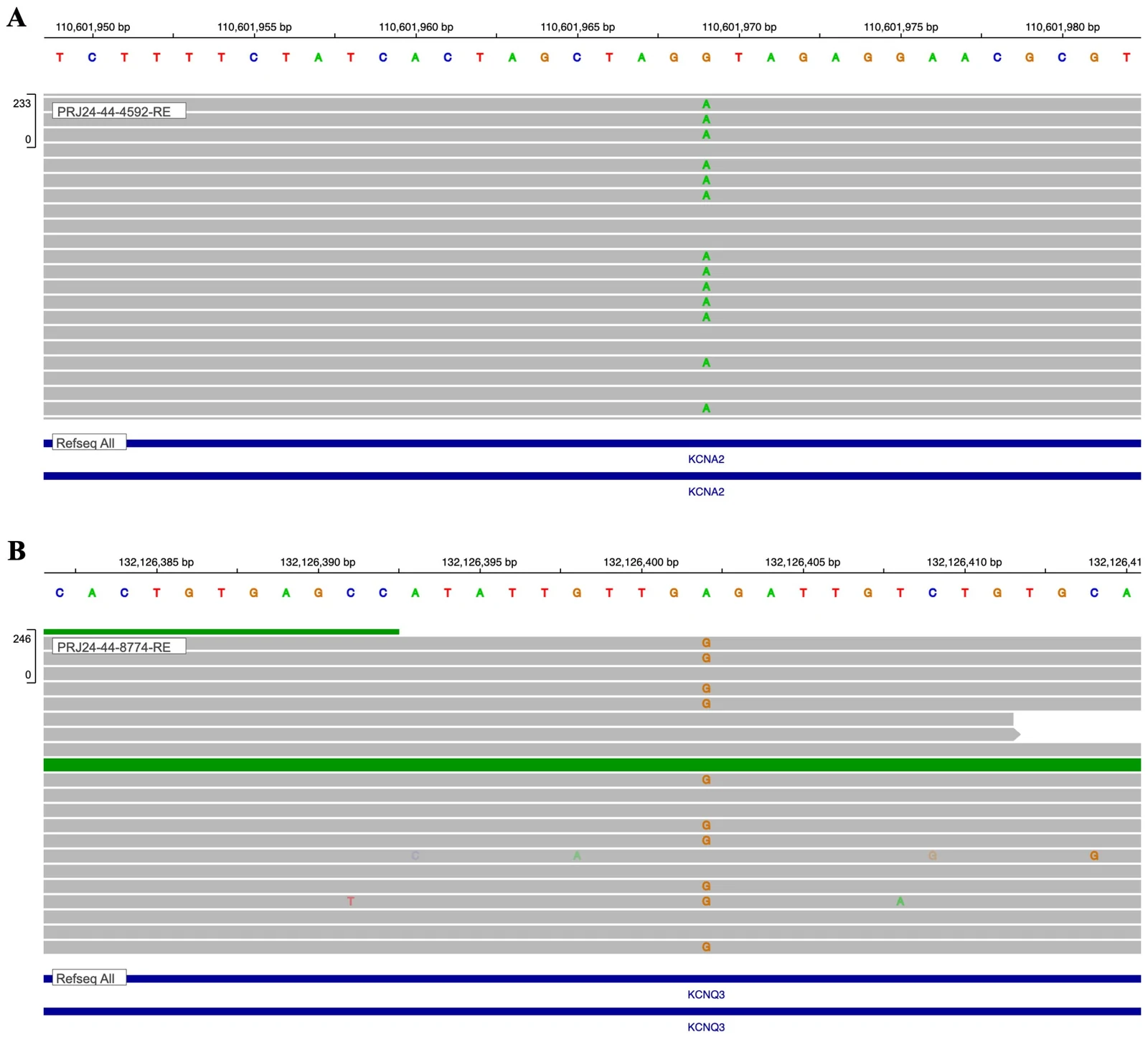
IGV illustrating the reads of the *KCNA2* gene chr1:110601969G>A *(KCNA2*:c.*1314C>T (**A**), and *KCNQ3* gene chr8:132126402A>G (*KCNQ3*:c.*2860T>C) (**B**) novel variations.

Nineteen variants were identified in *KCNA1* gene, predominantly 3′ UTR or synonymous variants, and all were classified as benign. One stop-gained variant (c.684T>C, p.Cys228=) was observed. Minor allele frequencies (MAFs) ranged from 0.01 to 0.50, indicating that most variants are common in the population. Six variants were detected in the *KCNA2* gene, mainly 3′ UTR and synonymous changes. All were benign, except for *KCNA2*:c.*1314C>T, a novel VUS that is not present in population databases (PM2). *KCNA2*:c.*1314C>T is shown in Figure 1. Thirty-one variants were identified in the *KCNQ1* gene, including intronic, non-coding transcript exon, 3′ UTR, and synonymous variants. Most were benign, except *KCNQ1:c.976G>A, classified as VUS (PM2), and rare missense variant *KCNQ1*:c.1179G>T (p.Lys393Asn), classified as benign. Among the analyzed *KCNQ1* rs34601797 variant, the intronic duplications c.1733-363dup and c.1733-364_1733-363dup were detected, both classified as benign and present at similar allele frequencies in the studied group. Fifteen variants were observed in the *KCNQ2* gene, including 3′ UTR, intronic, synonymous, and missense variants. Most were benign; however, *KCNQ2*:c.2613G>T (p.Arg871Ser) and *KCNQ2*:c.1148+62T>G were VUS with very low MAF (<0.01, PM2). Rare missense variants like *KCNQ2*:c.2065A>C (p.Ile689Leu) may have functional relevance. Thirty-nine variants were identified in *KCNQ3* gene, primarily located in the 3′ UTR and introns, with most being benign. Two variants, KCNQ3:c.*6282A>G and novel KCNQ3:c.*2860T>C, were classified as VUS. *KCNQ3*:c.*2860T>C is shown in Figure 1. Ten variants were detected in *KCNV2*, including 5′ UTR, 3′ UTR, synonymous, and missense variants. Most were benign; *KCNV2*:c.180C>T (p.Asp60=) was likely benign (PM2), and *KCNV2*:c.1597C>G (p.Leu533Val), a rare missense variant, may have functional significance (Table 1).

Six variants of VUS were observed, including *KCNA2* (c.*1314C>T), *KCNQ1* (c.*976G>A), *KCNQ2* (c.2613G>T p.Arg871Ser and c.1148+62T>G), and *KCNQ3* (c.*6282A>G and c.*2860T>C). Two novel variants were identified in our study group, *KCNA2*:c.*1314C>T and *KCNQ3*:c.*2860T>C. Both were located in the 3′ UTR region and classified as variants of VUS according to ACMG guidelines, supported by the PM2 criterion due to their absence in population databases (Figure 1A,B).

In addition to previously reported benign and likely benign variants, two novel variants were identified in our cohort: *KCNA2:c.1314C>T and *KCNQ3:c.2860T>C. Both were located in the 3′ UTR region and classified as variants of VUS according to ACMG guidelines, supported by the PM2 criterion due to their absence in population databases.

The selected variants within Kv genes were statistically analyzed between the drug-responsive and drug-persistent patient groups. No statistically significant differences were found in the distribution of the *KCNA1* gene (rs1048500T>C, rs2227910G>C, rs4766310C>T, and rs4766309T>A), the *KCNQ1* gene (rs8234A>G, rs2237895A>C), *KCNQ2* (rs1801475T>G, rs3746372C>A), the *KCNQ3* gene (rs112550767C>CCTGT, rs10095295T>A, and rs10108362A>G) or the *KCNV2* gene (rs7029012C>G, rs10967705C>G) between drug-responsive and drug-persistent epilepsy patients.

Overall, most identified variants across the six ion channel genes were common and benign, primarily located in UTRs, intronic, or synonymous regions. Rare or novel variants classified as VUS were detected in *KCNA2*, *KCNQ1*, *KCNQ2*, and *KCNQ3*, suggesting potential relevance for epileptic phenotypes that warrant further functional investigation.

### 2.2. Haplotype Analysis

Common variations of the *KCNA1* (rs1048500, rs2227910, rs4766310), *KCNQ1* (rs2237895, rs8234), *KCNQ2* (rs3746372, rs1801475), *KCNQ3* (rs10095295, rs10108362), and *KCNV2* (rs7029012, rs10967705) genes’ haplotypes were analyzed for association with epilepsy as sets of one, two, or three alleles. The chromosomal locations of the genes were identified as follows: *KCNQ1* at 11p15.5, *KCNQ2* at 20q13.33, *KCNQ3* at 8q24, *KCNA1* at 12p13.32, and *KCNV2* at 9q34.3. Haplotype analysis was performed separately for each gene, as the genes are located on different chromosomes.

**Figure 2:**
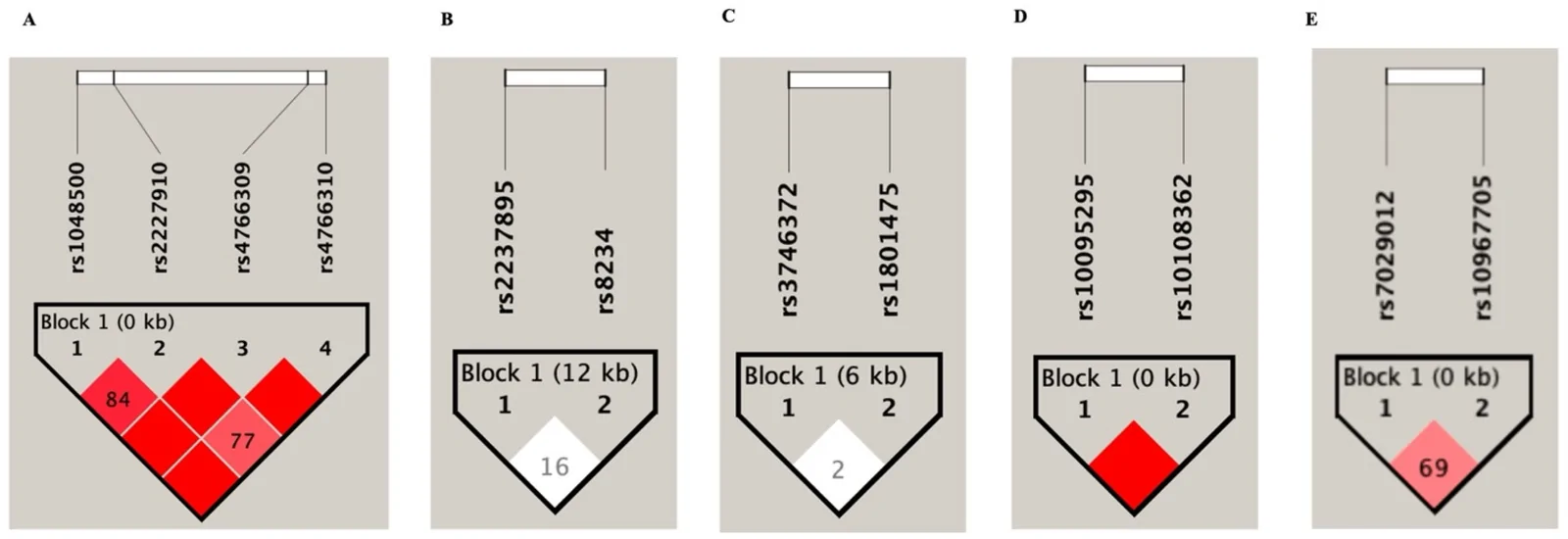
*KCNA1*, (**A**); *KCNQ1*, (**B**); *KCNQ2*, (**C**); *KCNQ*3, (**D**); and *KCNV2*, (**E**) genes common variations LD plots.

Table 2 lists the contig positions and marker numbers of polymorphisms in the haplotype analysis of the *KCNA1*, *KCNQ1*, *KCNQ2*, *KCNQ*3, and *KCNV2* genes. The LD analysis schemes were given to define haplotype blocks across the studied SNPs, as shown in Figure 2. A strong LD *KCN1A* gene block was identified between rs1048500, rs2227910, rs4766309, and rs4766310, with D′ values ranging from 0.77 to 0.84 suggesting strong allelic correlation within this region. High correlation was found between rs1048500 and rs2227910 (L′ 84%), between rs2227910 and rs4766310 (LD 77%), and between rs47666309 and rs4766310 (LD 100%). Among the polymorphisms, difference between rs1048500 and rs2227910 (D′ = 0.84; LOD = 6.45; r2 = 0.671) and linkage disequilibrium between rs2227910 and rs4766310 (D′ = 0.77; LOD = 5.72; r2 = 0.607) and between rs47666309 and rs4766310 (D′ = 1; LOD = 4.94; r2 = 0.51) have been revealed. This suggests a strong genetic linkage between these markers, which are often inherited together. However, high LD does not necessarily indicate that these SNPs are associated with the epilepsy drug response.

The LD plots of the analyzed SNPs are shown in Figure 2. The numbers in the squares represent D′ values. Red color indicates strong LD, while lighter colors represent weaker LD. Distinct haplotype blocks were defined using the Gabriel et al. method [21].

This block was subsequently included in haplotype association testing. Additionally, a strong LD was identified between *KCNQ3* rs10095295 and rs10108362 within the haplotype block. In contrast, rs2237895 and rs8234 *KCNQ1* showed weak LD (D′ = 0.16), whereas rs7029012 and rs10967705 *KCNV2* showed moderate linkage disequilibrium (D′ = 0.69, LOD = 3.88, r^2^ = 0.449). Although the D′ value indicates moderate linkage disequilibrium between these variants, the corresponding r^2^ value suggests a moderate level of allelic correlation. In contrast, rs3746372 and rs1801475 *KCNQ2* exhibited very weak LD (D′ = 0.02). Haplotype blocks were defined according to the Gabriel et al. method based primarily on D′ values, while r^2^ values were used to complement the interpretation of marker correlation.

In the analysis performed using the Haploview program, no statistically significant difference was found in the single or combined allele results of the *KCNA1*, *KCNQ1*, *KCNQ2*, and *KCNQ*3 genes. No single-marker associations reached statistical significance in the *KCNV2* gene.

**Figure 3:**
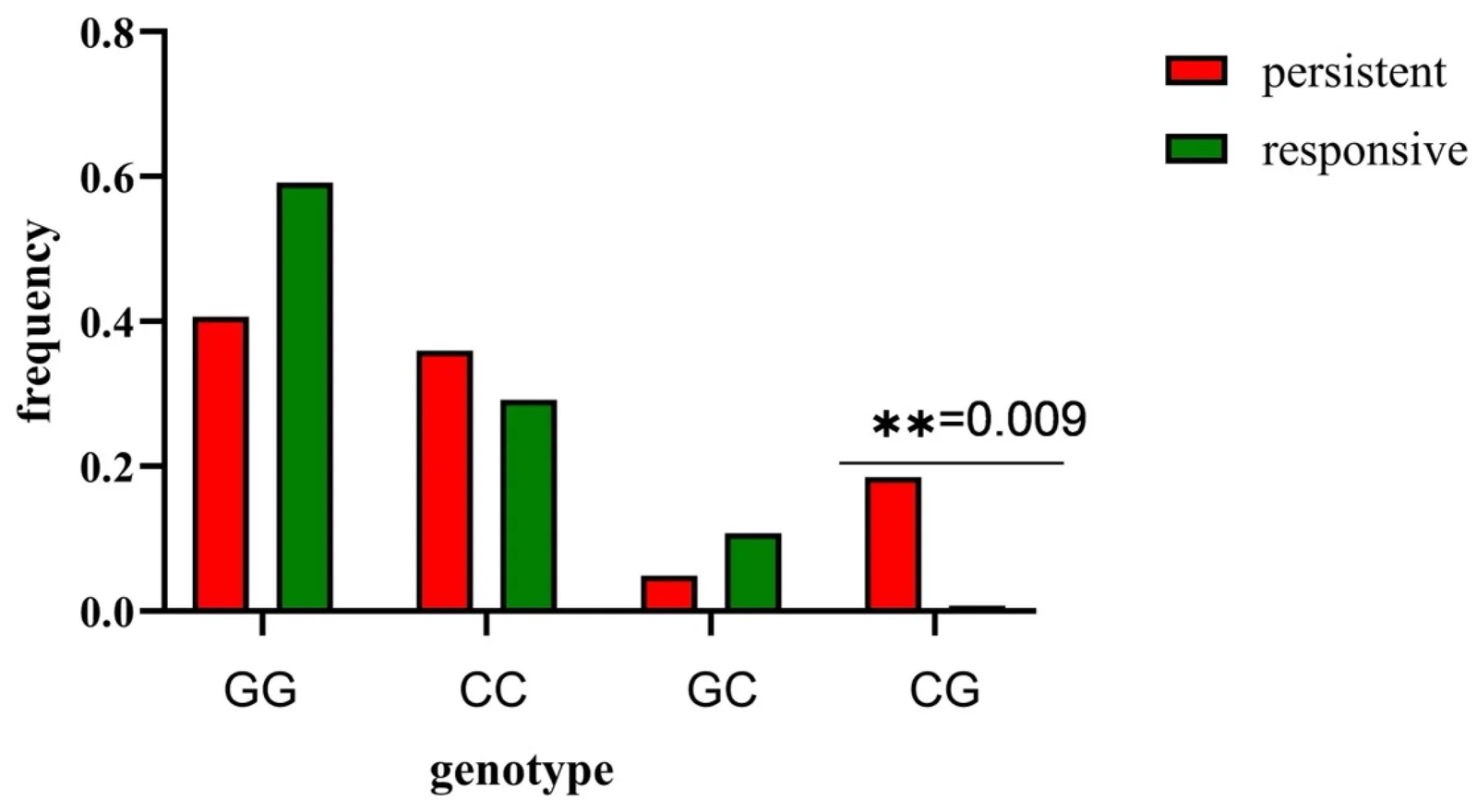
Distribution of the *KCNV2* gene rs7029012 and rs10967705 haplotypes in drug-persistent and drug-responsive epilepsy patients.

Haplotype analysis of *KCNV2* gene identified four major haplotypes (GG, CC, GC, CG) and a single-marker haplotype with distributions between drug-persistent and drug-responsive epilepsy patients (Table 3). The GG haplotype was the most common overall (0.526) and was more frequent in drug-responsive patients (0.592) compared to drug-persistent patients (0.406); however, the difference did not reach statistical significance (χ^2^ = 1.972, *p* = 0.160). Interestingly, the CG haplotype comprising the *KCNV2* rs7029012 and rs10967705 variants was observed more frequently in drug-persistent epilepsy patients (0.185) than in drug-responsive patients (0.008) (χ^2^ = 6.756, *p* = 0.009, BH-FDR q = 0.036) (Figure 3).

Haplotype analysis of the *KCNV2* gene revealed that the combination of rs7029012 (c.-42C>G, 5′ UTR variant) and rs10967705 (c.183C>G, synonymous variant, p.Gly61=) statistical significance in relation to the studied phenotype (Table 3). Both variants are individually classified as benign and do not alter the amino acid sequence or coding function. While the observed suggests a potential modulatory effect of this haplotype, the association remains inconclusive and warrants further investigation in larger cohorts to clarify its possible impact on gene regulation or disease susceptibility.

## 3. Discussion

The present pilot pharmacogenetic study comprehensively evaluated genetic variation in six voltage-gated potassium channel genes in Turkish patients with epilepsy using targeted next-generation sequencing. Although most of the 181 identified variants were classified as benign or likely benign according to ACMG criteria and no individual variant was significantly associated with antiseizure medication (ASM) response, several findings deserve attention. We identified six variants of VUS, including two previously unreported variants in *KCNA2* and *KCNQ3*, expanding the current spectrum of potassium channel gene variation in epilepsy. Most importantly, haplotype analysis revealed that the *KCNV2* CG haplotype (rs7029012-rs10967705) was significantly enriched in patients with drug-persistent epilepsy compared with drug-responsive patients. These findings suggest that while single Kv gene variants may have limited predictive value for ASM response, specific haplotypes within potassium channel genes may contribute to pharmacoresistance and represent promising pharmacogenetic biomarkers that warrant further investigation.

Voltage-gated potassium channels are essential regulators of neuronal excitability and play a central role in stabilizing the resting membrane potential, making them attractive therapeutic targets in epilepsy [22,23]. Despite substantial advances in antiseizure medications (ASMs), drug-resistant epilepsy remains a major clinical challenge, emphasizing the need for therapies targeting novel molecular pathways. Pharmacological activation of potassium channels, particularly Kv7 (KCNQ2/KCNQ3), Kv1.1, KATP, and GIRK2 channels, has been shown to suppress neuronal hyperexcitability and reduce seizure susceptibility [3,5,24]. In the present study, although no individual potassium channel variant was significantly associated with ASM response, the identification of a *KCNV2* haplotype enriched in drug-persistent patients supports the concept that inherited variation within potassium channel genes may influence treatment response. These findings suggest that the cumulative effects of multiple genetic variants, rather than individual polymorphisms alone, may contribute to pharmacoresistance and further reinforce potassium channels as promising targets for precision medicine in epilepsy.

Voltage-gated potassium channel genes, including *KCNA1*, *KCNA2*, *KCNQ1*, *KCNQ2*, *KCNQ3*, and *KCNV2*, have been implicated in epilepsy susceptibility; however, their contribution to antiseizure medication (ASM) response remains incompletely understood. In the present study, no significant associations were observed between individual variants in these genes and treatment response among Turkish patients with epilepsy. These findings suggest that common polymorphisms within Kv channel genes alone may have limited predictive value for ASM responsiveness. Similarly, Qu et al. found no significant associations between variants in *KCNA1*, *KCNA2*, or *KCNV2* and either epilepsy susceptibility or drug resistance in patients with genetic generalized epilepsy, indicating that these genes may not independently determine disease risk or treatment outcome [23]. Likewise, Al-Eitan et al. reported that, among several polymorphisms evaluated in *KCNA1*, *KCNA2*, and *KCNV2*, only the *KCNA2* variant rs3887820 was associated with an increased risk of generalized myoclonic seizures, whereas no significant associations were identified for *KCNA1* or *KCNV2* with either epilepsy susceptibility or ASM response [25]. Collectively, these findings suggest that the influence of Kv channel genes on pharmacoresistance is likely modest and may depend on the combined effects of multiple variants, haplotypes, or interactions with other genetic and environmental factors rather than on single polymorphisms alone.

Common genetic variants often exert their effects indirectly by serving as markers for nearby functional variants through linkage disequilibrium rather than by directly altering protein function [26]. Accordingly, haplotype analysis may capture the combined effects of multiple linked variants that are not detectable in single-variant association analyses. Although no individual SNP was significantly associated with ASM response in our cohort, the *KCNV2* CG haplotype (rs7029012-rs10967705) was significantly more frequent in patients with drug-persistent epilepsy than in drug-responsive patients (18.5% vs. 0.8%, *p* = 0.009, BH-FDR *q* = 0.036). This finding suggests that the *KCNV2* CG haplotype may represent a potential pharmacogenetic marker of ASM resistance. Because *KCNV2* encodes the modulatory Kv8.2 potassium channel subunit, genetic variation within this locus may influence neuronal excitability; however, this hypothesis requires experimental confirmation through functional electrophysiological studies. However, as both constituent variants are currently classified as benign, the observed association is more likely to reflect linkage with unidentified functional variants or regulatory elements than a direct biological effect. Functional studies, particularly electrophysiological characterization of the rs7029012-rs10967705 haplotype, are required to determine whether these variants alter channel properties, neuronal excitability, or antiseizure medication responsiveness. As both variants are currently classified as benign, the observed association may reflect linkage disequilibrium with nearby functional regulatory variants rather than a direct biological effect.

A notable finding of the present study was the strong linkage disequilibrium (LD) observed between *KCNV2* rs7029012 and rs10967705 (D′ = 0.82), supporting the presence of a haplotype block within this genomic region. This observation is consistent with the findings of Qu et al., who also reported significant LD between these two variants (D′ = 0.70) and suggested that rs10967705 may be associated with susceptibility to childhood absence epilepsy [23]. However, after Bonferroni correction for multiple testing, no variants in *KCNA1*, *KCNA2*, or *KCNV2* remained significantly associated with epilepsy risk or drug resistance [23]. Together with our findings, these results indicate that, although individual variants may have limited predictive value, haplotype-based analyses may better capture the combined genetic effects influencing epilepsy susceptibility and ASM response. Nevertheless, the exploratory nature of our study and the limited sample size warrant cautious interpretation, and independent replication in larger cohorts is required.

The identification of genetic determinants of pharmacoresistance is a prerequisite for the implementation of precision medicine in epilepsy [27]. Targeted next-generation sequencing enabled the comprehensive characterization of genetic variation across six voltage-gated potassium channel genes, identifying 181 variants, including rare and previously unreported alterations. Although individual variants were not significantly associated with ASM response in this cohort, these findings underscore the genetic heterogeneity of potassium channel genes in epilepsy and highlight the value of tNGS for discovering candidate variants that warrant functional validation in larger pharmacogenetic studies.

Integrating pharmacogenetic information into clinical practice has the potential to improve ASM selection and facilitate precision medicine approaches in epilepsy. In the present study, the majority of variants identified in voltage-gated potassium channel genes were classified as benign or likely benign, consistent with their relatively high population frequencies. Nevertheless, the identification of several VUS, including two previously unreported variants, expands the current spectrum of genetic variation in epilepsy. Although these variants were detected in both drug-responsive and drug-persistent patients and their clinical significance remains uncertain, they represent promising candidates for future functional and genotype–phenotype studies aimed at clarifying their potential role in ASM response and epilepsy pathogenesis.

Although no statistically significant differences were observed, the numerically higher frequency of VUS in the drug-persistent group raises the possibility that rare variants in potassium channel genes may influence ASM response. However, because these variants have not been functionally characterized, it remains unclear whether they alter channel function or contribute to pharmacoresistance. Further functional and genotype–phenotype studies are needed to clarify their biological and clinical relevance.

Consistent with previous pharmacogenetic studies, our findings suggest that individual genetic variants in potassium channel genes have a limited impact on ASM response across the heterogeneous range of currently available antiseizure medications [26]. This observation is biologically plausible, as most ASMs exert their therapeutic effects through mechanisms other than the direct modulation of potassium channels. Nevertheless, our exploratory haplotype analysis indicates that the combined effects of linked genetic variants, rather than individual polymorphisms, may contribute to interindividual differences in treatment response. As potassium channel-targeting agents continue to emerge, such as selective Kv7 channel openers, the relationship between potassium channel gene variation and response to these mechanism-specific therapies warrants further investigation. Thus, potassium channel modulators remain promising candidates for future precision medicine approaches in epilepsy [26].

The majority of the variants identified in this cohort were classified as benign or likely benign according to ACMG criteria, suggesting limited immediate clinical relevance. However, the identification of several variants classified as VUS, including two previously unreported variants in *KCNA2* and *KCNQ3*, expands the current spectrum of potassium channel gene variation in epilepsy. Although the biological and clinical relevance of these variants remains uncertain, they represent potential candidates for future functional studies and genotype–phenotype correlation analyses to clarify their possible contribution to epilepsy susceptibility and ASM response.

The majority of the variants identified in this cohort were classified as benign or likely benign according to ACMG criteria, suggesting that they are unlikely to have substantial functional or clinical significance. However, the identification of several variants of VUS, including two previously unreported variants in *KCNA2* and *KCNQ3*, expands the current spectrum of potassium channel gene variation in epilepsy. Although the clinical relevance of these variants remains uncertain, they represent important candidates for future functional studies and genotype–phenotype correlation analyses to clarify their potential contribution to epilepsy susceptibility and ASM response.

Although the clinical relevance of most identified variants remains limited, these findings expand the current spectrum of potassium channel gene variation. Particularly, the novel variants may provide valuable insights for future genotype–phenotype correlation studies in epilepsy and highlight the need for functional validation studies to elucidate their biological effects.

### Limitations and Strengths

This study has several limitations that should be acknowledged. First, the relatively small sample size limited the statistical power to detect modest genetic effects and increased the risk of false-positive or false-negative findings. Therefore, the observed association between the *KCNV2* haplotype and ASM response should be interpreted cautiously as exploratory and hypothesis-generating rather than confirmatory.

Second, the single-center design and inclusion of only Turkish patients may limit the generalizability of our findings to other ethnic populations. Although epilepsy subtype was recorded and did not differ significantly between the drug-responsive and drug-persistent groups, disease duration and seizure frequency were not available for all participants and therefore could not be incorporated into the association analyses. Third, patients were not stratified according to the specific ASMs received. Therefore, it was not possible to determine whether the observed haplotype association is related to resistance to particular ASMs or reflects a more general association with drug-resistant epilepsy. Future studies with larger, more homogeneous treatment groups and comprehensive clinical phenotyping are needed to clarify potential drug-specific genetic effects and genotype–phenotype relationships. Finally, although six VUS and two novel variants were identified, functional validation studies, including in vitro or in vivo assays, were not performed to assess their biological effects. Therefore, the functional relevance of these variants and the biological implications of the identified haplotypes remain to be elucidated. In particular, electrophysiological validation was beyond the scope of the present clinical pharmacogenetic study and should be addressed in future mechanistic investigations. Larger, multicenter studies involving ethnically diverse populations, together with functional investigations, are required to validate these findings and clarify the mechanistic role of potassium channel gene variation in ASM response.

Despite these limitations, this study has several strengths. To our knowledge, this is the first targeted next-generation sequencing study to comprehensively evaluate the *KCNA1*, *KCNA2*, *KCNQ1*, *KCNQ2*, *KCNQ3*, and *KCNV2* genes in relation to ASM response in a Turkish epilepsy cohort. The use of high-depth targeted sequencing enabled the detection of both common and rare variants, including two previously unreported variants, while haplotype analysis provided additional pharmacogenetic insights beyond conventional single-variant analyses. These findings provide a valuable foundation for future precision medicine studies in epilepsy.

## 4. Methods

### 4.1. Study Population

This pilot exploratory pharmacogenetic study was conducted in accordance with the Declaration of Helsinki on medical research due to the involvement of a human study group. The Non-Interventional Clinical Research Ethics Committee of Istanbul University-Cerrahpaşa gave approval (approval number: 2025/428; date: 4 June 2025). Written informed consent was obtained from all participants.

Thirty-five patients with epilepsy receiving routine ASM treatment were screened at the Republic of Türkiye Ministry of Health, Sinan Sipahi Family Health Centre (Istanbul, Türkiye). Two patients did not meet the inclusion criteria and two declined participation, leaving 31 patients for the final analysis. Eligible participants were aged 18–75 years and had complete clinical and genetic data. The mean age of the participants was 45.52 ± 15.51 years. The mean age was 47.40 ± 18.28 years in the drug-responsive group and 42.51 ± 16.40 years in the drug-persistent group, with no significant difference between the groups (*p* = 0.647). Similarly, the sex distribution did not differ significantly between the two groups (*p* = 0.793). The study population received ASMs as either monotherapy or polytherapy. The treatment regimen most commonly included valproic acid (VPA), levetiracetam (LEV), carbamazepine (CBZ) and lamotrigine (LTG), while topiramate (TPM), lacosamide (LCM), oxcarbazepine (OXC), phenytoin (PHT), and clonazepam (CLZ) were prescribed in a smaller proportion of patients. Epilepsy subtypes were classified as focal, generalized, or combined focal/generalized epilepsy, and no significant difference in subtype distribution was observed between the drug-responsive and drug-persistent groups (*p* = 0.220).

Patients were classified according to the International League Against Epilepsy (ILAE) criteria as drug-responsive (≥12 months seizure-free; *n* = 19) or drug-persistent (≥4 seizures/year despite treatment with ≥2 ASMs; *n* = 12) [28]. The mean age of the cohort was 43.2 ± 17.9 years, and 56.2% were male. No significant differences in age or sex were observed between the groups (*p* > 0.05).

Because this was a pilot study, the primary objective was hypothesis generation and identification of potential pharmacogenetic associations rather than confirmatory testing. Sample size estimation was performed using G*Power 3.1 based on the expected effect size for treatment response.

### 4.2. Blood Collection and DNA Isolation

Peripheral venous blood (10 mL) was collected into EDTA tubes during routine outpatient follow-up visits over a two-month period. Samples were stored at 4 °C until genomic DNA extraction.

Genomic DNA was isolated using the QIAamp DNA Micro Kit (Qiagen, Hilden, Germany; Cat. No. 56304) according to the manufacturer’s instructions. DNA concentration and purity were assessed using the Qubit 3.0 Fluorometer (Invitrogen, Carlsbad, CA, USA).

### 4.3. Targeted Next-Generation Sequencing

Targeted next-generation sequencing (tNGS) was performed for the coding regions and exon–intron boundaries of six potassium channel genes (*KCNQ1*, *KCNQ2*, *KCNQ3*, *KCNA1*, *KCNA2*, and *KCNV2*). Target regions were amplified by PCR using a MiniAmp Thermal Cycler (Thermo Fisher Scientific, Waltham, MA, USA).

Amplicon libraries were prepared using manually designed primers, indexed, pooled, and sequenced on the Illumina NovaSeq 6000 platform (Illumina Inc., San Diego, CA, USA). All target regions achieved a minimum sequencing depth of 100×.

### 4.4. Bioinformatic Analysis and Variant Interpretation

Raw sequencing reads underwent quality assessment using FastQC 0.11.9 followed by adapter trimming and quality filtering with Cutadapt 5.0. High-quality reads were aligned to the GRCh38 human reference genome and processed through the DRAGEN 4.3.6 analysis pipeline [29,30].

Variant annotation was performed using Ensembl Variant Effect Predictor (VEP) 115.2 and all prioritized variants were visually confirmed using the Integrative Genomics Viewer (IGV) 3.8.3.

Rare variants (minor allele frequency < 1% in gnomAD 4.1) with potential functional significance were prioritized and interpreted using the Ilyome Bioinformatics platform. Variant classification followed the American College of Medical Genetics and Genomics/Association for Molecular Pathology (ACMG/AMP) 2015 guidelines [31].

### 4.5. Statistical Analysis

Statistical analyses were performed using GraphPad Prism version 10.5.0. The normality of continuous variables was assessed using the Shapiro–Wilk test. Since age was not normally distributed, between-group comparisons were performed using the Mann–Whitney U test. Sex distribution was compared using the Chi-square test, while allele, genotype, and variant frequency comparisons were performed using Fisher’s exact test. Statistical significance was defined as *p* < 0.05. Allele and genotype frequencies were calculated, and the Hardy–Weinberg equilibrium (HWE) was assessed using the online HWE calculator developed by Michael H. Court. Linkage disequilibrium (LD) and haplotype analyses were performed using Haploview 4.1 software. To account for multiple comparisons, the Benjamini–Hochberg false discovery rate (BH-FDR) procedure was applied separately to the single-marker and haplotype association analyses. Adjusted *q*-values < 0.05 were considered statistically significant.

### 4.6. Safety Assessment

Study-related adverse events were prospectively monitored throughout follow-up using structured clinical questionnaires and routine laboratory evaluations. Participants were encouraged to report any unexpected events at any time. No study-related adverse events were observed.

## 5. Conclusions

This pilot pharmacogenetic study provides two principal findings. First, most variants identified in *KCNA1*, *KCNA2*, *KCNQ1*, *KCNQ2*, *KCNQ3*, and *KCNV2* were classified as benign or likely benign according to ACMG criteria. However, the identification of six variants of uncertain significance, including two previously unreported variants, expands the current spectrum of potassium channel gene variation in epilepsy and provides candidate variants for future functional studies.

Second, although no significant associations were observed for individual variants, the haplotype analysis identified a preliminary association between the KCNV2 rs7029012–rs10967705 CG haplotype and drug-resistant epilepsy, which remained significant after BH-FDR correction.

The findings of this pilot study should be interpreted in light of several limitations, including the relatively small sample size, the inclusion of only a Turkish population, the lack of a healthy control group, the absence of functional validation of the identified variants and haplotypes, and the inability to evaluate drug-specific genetic associations because patients were not stratified according to the specific antiseizure medications received. Consequently, it remains unclear whether the observed KCNV2 haplotype association is related to resistance to particular ASMs or reflects a more general association with drug-resistant epilepsy. Therefore, larger multicenter studies involving multi-ethnic populations, homogeneous treatment groups, healthy controls, and functional investigations are required to validate these findings, clarify the biological significance of the identified variants and haplotypes, and determine their potential role in antiseizure medication response. Although the identified KCNV2 haplotype represents a promising pharmacogenetic candidate, its biological relevance cannot be established without independent replication and functional electrophysiological studies.

## Figures and Tables

**Table 1:** A list of rare and common variants of *KCNQ1*, *KCNQ2*, *KCNQ*3, *KCNA1*, *KCNA2*, and *KCNV2* genes detected in epileptic patients.

Epileptic Patients								
	Gene Variant	dbSNP ID	HGVS GRCh38-Positions	Variation Type	ACMG Classification	Amino Acid Change	Highest Population MAF	Pathogenic Criteria
	***KCNA1***							
**1**	*KCNA1*:c.*1522C>G	rs140297443	chr12-4914388 C>G	3 prime UTR variant	Benign		0.07	
**2**	*KCNA1*:c.*1927A>T	rs7974559	chr12-4914793 A>T	3 prime UTR variant	Benign		0.50	
**3**	*KCNA1*:c.684T>C	rs1048500	chr12-4912062 T>C	stop gained	Benign	p.Cys228=	0.50	
**4**	*KCNA1*:c.*406C>T	rs4766311	chr12-4913272 C>T	3 prime UTR variant	Benign		0.50	
**5**	*KCNA1*:c.*3062T>C	rs10849174	chr12-4915928 T>C	3 prime UTR variant	Benign		0.50	
**6**	*KCNA1*:c.804G>C	rs2227910	chr12-4912182 G>C	synonymous variant	Benign	p.Thr268=	0.50	
**7**	*KCNA1*:c.1440T>A	rs4766309	chr12-4912818 T>A	synonymous variant	Benign	p.Thr480=	0.38	
**8**	*KCNA1*:c.*9G>A	rs4766310	chr12-4912875 G>A	3 prime UTR variant	Benign		0.50	
**9**	*KCNA1*:c.*2277_*2278del	rs397724146	chr12-4915137 GCC>G	3 prime UTR variant	Benign		0.50	
**10**	*KCNA1*:c.*5141G>A	rs41482147	chr12-4918007 G>A	3 prime UTR variant	Benign		0.25	
**11**	*KCNA1*:c.*1524A>G	rs79357862	chr12-4914390 A>G	3 prime UTR variant	Benign		0.25	
**12**	*KCNA1*:c.*4439C>T	rs12424292	chr12-4917305 C>T	3 prime UTR variant	Benign		0.39	
**13**	*KCNA1*:c.*2333G>A	rs2109420	chr12-4915199 G>A	3 prime UTR variant	Benign		0.25	
**14**	*KCNA1*:c.*1577A>T	rs79432803	chr12-4914443 A>T	3 prime UTR variant	Benign		0.01	
**15**	*KCNA1*:c.*4234A>G	rs61907267	chr12-4917100 A>G	3 prime UTR variant	Benign		0.03	
**16**	*KCNA1*:c.*2080G>A	rs147644839	chr12-4914946 G>A	3 prime UTR variant	Benign		0.01	
**17**	*KCNA1*:c.-134G>T	rs12817318	chr12-4911245 G>T	5 prime UTR variant	Benign		0.12	
**18**	*KCNA1*:c.*2596A>T	rs188377239	chr12-4915462 A>T	3 prime UTR variant	Benign		0.01	
**19**	*KCNA1*:c.*2174C>T	rs73050551	chr12-4915040 C>T	3 prime UTR variant	Benign		0.50	
	***KCNA2***							
**1**	*KCNA2*:c.*1379A>C	rs4839071	chr1-110601904 T>G	3 prime UTR variant	Benign		0.01	
**2**	*KCNA2*:c.*1045del	rs75305356	chr1-110602237 AT>A	3 prime UTR variant	Benign		0.18	
**3**	*KCNA2*:c.1026T>C	rs12407942	chr1-110602039 A>G	3 prime UTR variant	Benign	p.Asp342=	0.47	
**4**	*KCNA2*:c.*9294C>T	rs17026168	chr1-110593989 G>A	3 prime UTR variant	Benign		0.49	
**5**	*KCNA2*:c.1185G>C	rs78349687	chr1-110603598 C>G	synonymous variant	Benign	p.Ala395=	0.11	
**6**	*KCNA2*:c.*1314C>T	novel	chr1-110601969 G>A	3 prime UTR variant	VUS			PM2
	***KCNQ1***							
**1**	*KCNQ1*:c.1514+46A>G	rs760419	chr11-2662127 A>G	non-coding transcript exon variant	Benign		0.50	
**2**	*KCNQ1*:c.*932A>G	rs10798	chr11-2848935 A>G	3 prime UTR variant	Benign		0.49	
**3**	*KCNQ1*:c.1685+36A>G	rs163150	chr11-2776090 A>G	intron variant	Benign		0.47	
**4**	*KCNQ1*:c.*875A>G	rs8234	chr11-2848878 A>G	3 prime UTR variant	Benign		0.50	
**5**	*KCNQ1*:c.1732+43T>C	rs81204	chr11-2777075 T>C	intron variant	Benign		0.32	
**6**	*KCNQ1*:c.1794+13G>A	rs776674137	chr11-2778050 G>A	intron variant	Likely Benign		<0.01	PM2
**7**	*KCNQ1*:c.1795-11803A>C	rs2237895	chr11-2835964 A>C	intron variant	Benign		0.50	
**8**	*KCNQ1*:c.1795-11764G>A	rs60808706	chr11-2836003 G>A	intron variant	Benign		0.42	
**9**	*KCNQ1*:c.1733-357C>T	rs2741950	chr11-2777619 C>T	intron variant	Benign		0.50	
**10**	*KCNQ1*:c.1733-361_1733-360insA	rs71302039	chr11-2777615 G>GA	intron variant	Benign		0.50	
**11**	*KCNQ1*:c.1733-362A>G	rs2411343	chr11-2777614 A>G	intron variant	Benign		0.49	
**12**	*KCNQ1*:c.1733-363dup	**rs34601797**	chr11-2777611 C>CG	intron variant	Benign		0.49	
**13**	*KCNQ1*:c.1733-364_1733-363dup	**rs34601797**	chr11-2777611 C>CGG	intron variant	Benign		0.49	
**14**	*KCNQ1*:c.1795-29246C>T	rs2237892	chr11-2818521 C>T	intron variant	Benign		0.40	
**15**	*KCNQ1*:c.1515-55G>A	rs2075870	chr11-2768789 G>A	intron variant	Benign		0.25	
**16**	*KCNQ1*:c.1514+46A>G	rs760419	chr11-2662127 A>G	non-coding transcript exon variant	Benign		0.50	
**17**	*KCNQ1*:c.1638G>A	rs1057128	chr11-2776007 G>A	synonymous variant	Benign	p.Ser546=	0.42	PM2
**18**	*KCNQ1*:c.604+33C>G	rs774970104	chr11-2570787 C>G	intron variant	Likely Benign		<0.01	
**19**	*KCNQ1*:c.1394-39T>G	rs739502	chr11-2661922 T>G	non-coding transcript exon variant	Benign		0.50	
**20**	*KCNQ1*:c.1986C>T	rs11601907	chr11-2847958 C>T	stop gained	Benign	p.Tyr662=	0.32	
**21**	*KCNQ1*:c.1685+23G>A	rs190094645	chr11-2776077 G>A	intron variant	Benign		0.02	
**22**	*KCNQ1*:c.1733-302T>C	rs182093946	chr11-2777674 T>C	intron variant	Benign		0.03	
**23**	*KCNQ1*:c.1794+32G>T	rs41282928	chr11-2778069 G>T	intron variant	Benign		0.09	
**24**	*KCNQ1*:c.386+16523A>G	rs372562223	chr11-2462007 A>G	intron variant	Benign		0.08	
**25**	*KCNQ1*:c.386+16519_386+16522del	rs202218721	chr11-2462002 GTCCC>G	intron variant	Benign		0.08	
**26**	*KCNQ1*:c.*976G>A	rs191331870	chr11-2848979 G>A	3 prime UTR variant	VUS		0.01	PM2
**27**	*KCNQ1*:c.1514+18C>T	rs12577654	chr11-2662099 C>T	non-coding transcript exon variant	Benign		0.07	
**28**	*KCNQ1*:c.1590+14T>C	rs11024034	chr11-2768933 T>C	intron variant	Benign		0.19	
**29**	*KCNQ1*:c.1795-29208C>T	rs145839955	chr11-2818559 C>T	intron variant	Benign		0.03	
**30**	*KCNQ1*:c.*742G>A	rs114844136	chr11-2848745 G>A	3 prime UTR variant	Benign		0.09	
**31**	*KCNQ1*:c.1179G>T	rs12720457	chr11-2587620 G>T	missense variant	Benign	p.Lys393Asn	0.03	PM5
	***KCNQ2***							
**1**	*KCNQ2*:c.*5962G>T	rs3746372	chr20-63400682 C>A	3 prime UTR variant	Benign		0.46	
**2**	*KCNQ2*:c.*5853C>T	rs62208000	chr20-63400791 G>A	missense variant	Benign		0.17	
**3**	*KCNQ2*:c.1503C>G	rs1801545	chr20-63414925 G>C	synonymous variant	Benign	p.Ala501=	0.20	
**4**	*KCNQ2*:c.297-12345G>A	rs62208041	chr20-63459182 C>T	intron variant	Benign		0.14	
**5**	*KCNQ2*:c.388-26G>T	rs6062939	chr20-63445390 C>A	intron variant	Benign		0.45	
**6**	*KCNQ2*:c.627C>A	rs749602639	chr20-63444722 G>T	synonymous variant	Likely Benign	p.Ile209=	<0.01	PM2
**7**	*KCNQ2*:c.2339A>C	rs1801475	chr20-63406924 T>G	missense variant	Benign	p.Asn780Thr	0.50	PP2
**8**	*KCNQ2*:c.*5847G>A	rs143295292	chr20-63400797 C>T	3 prime UTR variant	Benign		0.03	
**9**	*KCNQ2*:c.2238T>A	rs1801471	chr20-63407025 A>T	synonymous variant	Benign	p.Pro746=	0.28	
**10**	*KCNQ2*:c.2613G>T	rs587780369	chr20-63406650 C>A	missense variant	VUS	p.Arg871Ser	<0.01	PM2
**11**	*KCNQ2*:c.1248-72C>T	rs12481298	chr20-63419744 G>A	intron variant	Benign		0.16	
**12**	*KCNQ2*:c.912C>T	rs2297385	chr20-63439613 G>A	synonymous variant	Benign	p.Phe304=	0.48	
**13**	*KCNQ2*:c.1148+62T>G	rs531814304	chr20-63431278 A>C	intron variant	VUS		0.01	PM2
**14**	*KCNQ2*:c.2065A>C	rs201701585	chr20-63407198 T>G	missense variant	Benign	p.Ile689Leu	0.02	PP2
**15**	*KCNQ2*:c.1632-18C>A	rs368910668	chr20-63413599 G>T	intron variant	Benign		<0.01	
**16**	*KCNQ2*:c.1248-33G>A	rs765619875	chr20-63419705 C>T	intron variant	Benign		<0.01	PM2
	***KCNQ3***							
**1**	*KCNQ3*:c.*3977G>A	rs2469628	chr8-132125285 C>T	3 prime UTR variant	Benign		0.50	
**2**	*KCNQ3*:c.*4746_*4747insACAG	rs112550767	chr8-132124515 C>CCTGT	3 prime UTR variant	Benign		0.29	
**3**	*KCNQ3*:c.*4958A>G	rs1437824	chr8-132124304 T>C	3 prime UTR variant	Benign		0.50	
**4**	*KCNQ3*:c.*5719C>T	rs11786417	chr8-132123543 G>A	3 prime UTR variant	Benign		0.24	
**5**	*KCNQ3*:c.*6238T>C	rs10108362	chr8-132123024 A>G	3 prime UTR variant	Benign		0.43	
**6**	*KCNQ3*:c.*6282A>G	rs10095295	chr8-132122980 T>C	3 prime UTR variant	VUS		0.43	PM2
**7**	*KCNQ3*:c.*6632T>C	rs9297840	chr8-132122630 A>G	3 prime UTR variant	Benign		0.43	
**8**	*KCNQ3*:c.*7506C>T	rs11785257	chr8-132121756 G>A	3 prime UTR variant	Benign		0.24	
**9**	*KCNQ3*:c.*3032A>G	rs2469626	chr8-132126230 T>C	3 prime UTR variant	Benign		0.50	
**10**	*KCNQ3*:c.*3597G>A	rs1025436	chr8-132125665 C>T	3 prime UTR variant	Benign		0.50	
**11**	*KCNQ3*:c.*6812A>G	rs7815106	chr8-132122450 T>C	3 prime UTR variant	Benign		0.43	
**12**	*KCNQ3*:c.*5932A>C	rs2436130	chr8-132123330 T>G	3 prime UTR variant	Benign		0.47	
**13**	*KCNQ3*:c.*6340G>A	rs2469629	chr8-132122922 C>T	3 prime UTR variant	Benign		0.47	
**14**	*KCNQ3*:c.*6874C>G	rs2436129	chr8-132122388 G>C	3 prime UTR variant	Benign		0.47	
**15**	*KCNQ3*:c.*6956A>G	rs2469630	chr8-132122306 T>C	3 prime UTR variant	Benign		0.50	
**16**	*KCNQ3*:c.*7033T>C	rs2436128	chr8-132122229 A>G	3 prime UTR variant	Benign		0.47	
**17**	*KCNQ3*:c.*7143A>G	rs2436125	chr8-132122119 T>C	3 prime UTR variant	Benign		0.47	
**18**	*KCNQ3*:c.*7221C>T	rs2436124	chr8-132122041 G>A	3 prime UTR variant	Benign		0.50	
**19**	*KCNQ3*:c.*8148G>A	rs1437822	chr8-132121114 C>T	3 prime UTR variant	Benign		0.47	
**20**	*KCNQ3*:c.*3035A>G	rs2469627	chr8-132126227 T>C	3 prime UTR variant	Benign		0.46	
**21**	*KCNQ3*:c.1700+29G>A	rs2469515	chr8-132137856 C>T	intron variant	Benign		0.46	
**22**	*KCNQ3*:c.*2166A>G	rs35279095	chr8-132127096 T>C	3 prime UTR variant	Benign		0.08	
**23**	*KCNQ3*:c.*6446del	rs35153843	chr8-132122815 GT>G	3 prime UTR variant, deletion	Benign		0.07	
**24**	*KCNQ3*:c.*7464C>A	rs35604597	chr8-132121798 G>T	3 prime UTR variant	Benign		0.07	
**25**	*KCNQ3*:c.*7075A>G	rs2436127	chr8-132122187 T>C	3 prime UTR variant	Benign		0.47	
**26**	*KCNQ3*:c.*7119C>T	rs2436126	chr8-132122143 G>A	3 prime UTR variant	Benign		0.47	
**27**	*KCNQ3*:c.387-983C>A	rs58417923	chr8-132187164 G>T	intron variant	Benign		0.15	
**28**	*KCNQ3*:c.*7131G>A	rs75865310	chr8-132122131 C>T	3 prime UTR variant	Benign		0.11	
**29**	*KCNQ3*:c.*1050A>C	rs76720699	chr8-132128212 T>G	3 prime UTR variant	Benign		0.10	
**30**	*KCNQ3*:c.*3823G>A	rs17651980	chr8-132125439 C>T	3 prime UTR variant	Benign		0.03	
**31**	*KCNQ3*:c.*6831G>A	rs529301177	chr8-132122431 C>T	3 prime UTR variant	Benign		0.03	
**32**	*KCNQ3*:c.933+25T>C	rs17575971	chr8-132175428 A>G	intron variant	Benign		0.12	
**33**	*KCNQ3*:c.1236-64C>T	rs17653354	chr8-132163558 G>A	intron variant	Benign		0.12	
**34**	*KCNQ3*:c.1071C>G	rs17575754	chr8-132172667 G>C	synonymous variant	Benign	p.Leu357=	0.12	
**35**	*KCNQ3*:c.732T>C	rs41272387	chr8-132180202 A>G	synonymous variant	Benign	p.Gly244=	0.12	
**36**	*KCNQ3*:c.660T>C	rs41272389	chr8-132180274 A>G	synonymous variant	Benign	p.Asn220=	0.12	
**37**	*KCNQ3*:c.*2860T>C	novel	chr8-132126402 A>G	3 prime UTR variant	VUS			PM2
**38**	*KCNQ3*:c.1140+77T>A	rs3889950	chr8-132172521 A>T	intron variant	Benign		0.04	
**39**	*KCNQ3*:c.387-1068G>A	rs71526247	chr8-132187249 C>T	intron variant	Benign		0.03	
**40**	*KCNQ3*:c.1241A>G	rs2303995	chr8-132163489 T>C	missense variant	Benign	p.Glu414Gly	0.20	
	***KCNV2***							
**1**	*KCNV2*:c.-42C>G	rs7029012	chr9-2717698 C>G	5 prime UTR variant	Benign		0.46	
**2**	*KCNV2*:c.183C>G	rs10967705	chr9-2717922 C>G	synonymous variant	Benign	p.Gly61=	0.48	
**3**	*KCNV2*:c.795C>G	rs12237048	chr9-2718534 C>G	synonymous variant	Benign	p.Ala265=	0.49	
**4**	*KCNV2*:c.-64T>G	rs11793555	chr9-2717676 T>G	5 prime UTR variant	Benign		0.40	
**5**	*KCNV2*:c.1083A>G	rs142744007	chr9-2718822 A>G	synonymous variant	Benign	p.Gln361=	0.03	
**6**	*KCNV2*:c.-15C>T	rs181712064	chr9-2717725 C>T	5 prime UTR variant	Benign		0.08	
**7**	*KCNV2*:c.180C>T	rs1403529555	chr9-2717919 C>T	missense variant	Likely Benign	p.Asp60=	<0.01	PM2
**8**	*KCNV2*:c.*6T>C	rs41306094	chr9-2729733 T>C	3 prime UTR variant	Benign		0.23	
**9**	*KCNV2*:c.1386C>T	rs41312842	chr9-2729475 C>T	synonymous variant	Benign	p.Asp462=	0.16	
**10**	*KCNV2*:c.1597C>G	rs12352254	chr9-2729686 C>G	missense variant	Benign	p.Leu533Val	0.43	

MAF: Minor Allele Frequency. Highest minor allele frequency observed in any population including 1000 Genomes Phase 3, ESP and gnomAD. Markings in bold indicate different genetic variations of the same polymorphism. Gene variant, position, ACMG classification, amino acid change, and pathogenic criteria taken from Franklin Bioinformatics. Variation type and highest population for MAF are taken from Ensembl. Pathogenicity was evaluated based on the ACMG guidelines. PM2 (moderate): absent or very rare in population databases (gnomAD, etc.). PP2 (supporting): missense variants are rare in this gene, and loss-of-function is a known disease mechanism. PP5 (supporting): reported as pathogenic by a reputable source.

**Table 2:** Common *KCNA1*, *KCNQ1*, *KCNQ2*, *KCNQ*3, and *KCNV2* gene variations in haplotype analysis.

Gene	Marker	Polymorphism	Allele Change	Contig Position	MAF
*KCN1A*	Marker 1	rs1048500	C>T	4912062	0.466
	Marker 2	rs2227910	C>G	4912182	0.448
	Marker 3	rs47663309	T>A	4912818	0.293
	Marker 4	rs47663310	T>C	4912875	0.448
*KCNQ1*	Marker	Polymorphism	Allele Change	Contig Position	MAF
	Marker 1	rs2237895	A>C	2835964	0.345
	Marker 2	rs8234	A>G	2848878	0.362
*KCNQ2*	Marker	Polymorphism	Allele Change	Contig Position	MAF
	Marker 1	rs3746372	C>A	63400682	0.345
	Marker 2	rs1801475	T>G	63406924	0.345
*KCNQ3*	Marker	Polymorphism	Allele Change	Contig Position	MAF
	Marker 1	rs10095295	T>A	132122980	0.293
	Marker 2	rs10108362	A>G	132123024	0.293
*KCNQV2*	Marker	Polymorphism	Allele Change	Contig Position	MAF
	Marker 1	rs7029012	G>C	35033920	0.379
	Marker 2	rs10967705	G>C	35034035	0.431

MAF: minor allele frequency.

**Table 3:** Single-marker and haplotype association analysis of KCNV2 variants.

Frequencies							
Marker	Haplotype	Overall	Drug-Persistent	Drug-Responsive	Chi Square	*p* Value	BH-FDR q-Value
Single marker							
M1	C		0.545	0.300	3.604	0.057	0.114
M2	C		0.409	0.400	0.005	0.944	0.944
Haplotype							
M1+M2	GG	0.526	0.406	0.592	1.972	0.160	0.320
M1+M2	CC	0.316	0.360	0.292	0.307	0.579	0.579
M1+M2	GC	0.087	0.049	0.108	0.629	0.427	0.569
M1+M2	CG	0.071	0.185	0.008	6.756	0.009	0.036

BH-FDR-adjusted q-values are shown for multiple comparisons performed separately in the single-marker and haplotype analyses.

## Data Availability

The data supporting the findings of this study can be obtained from the corresponding author upon reasonable request.
